# Leukocytoclastic vasculitis as a rare dermatologic manifestation of Crohn’s disease mimicking cellulitis: a case report

**DOI:** 10.1186/s12876-020-01371-3

**Published:** 2020-07-29

**Authors:** Meredith Buck, Igor Dumic, Wendy McDermott, Charles Nordstrom, Samarth Dawan, Andrew Virata, Scott Martin, Ann Hudson, Tamara Milovanovic, Terri Nordin

**Affiliations:** 1 Mayo Clinic Family Medicine Residency – Eau Claire Program, Eau Claire, WI USA; 2 Mayo Clinic Alix College of Medicine and Science, Rochester, MN USA; 3 Division of Hospital Medicine, Mayo Clinic Health System, Eau Claire, WI USA; 4 Icahn School of Medicine at Mount Sinai, New York City, NY USA; 5 Department of Pathology, Mayo Clinic Health System, Eau Claire, WI USA; 6 Department of Gastroenterology, Clinical Center of Serbia, Belgrade, Serbia; 7 Department of Family Medicine, Mayo Clinic Health System, Eau Claire, WI USA

**Keywords:** Leukocytoclastic vasculitis, Crohn’s disease, Dermatologic manifestation, Cellulitis, Inflammatory bowel disease, Ustekinumab, Levofloxacin, Case report

## Abstract

**Background:**

Leukocytoclastic vasculitis (LCV) is an immune-complex mediated vasculitis characterized by neutrophilic inflammation and nuclear debris in post capillary venules. LCV is a rare dermatologic manifestation of Crohn’s disease (CD) and may occur with the onset of the disease or any time after the diagnosis including the period of exacerbation.

**Case presentation:**

We present a 70 year old woman with history of psoriasis and treatment refractory CD requiring monoclonal antibody therapy with ustekinumab. One month prior to the current admission, she developed abdominal pain, worsening diarrhea and was diagnosed with CD exacerbation for which she was given ustekinumab. While her abdominal symptoms mildly improved with ustekinumab, she developed new bilateral lower extremity rash initially treated with levofloxacin for presumed cellulitis. The rash consisted of mild erythematous, non-scaling patches with scattered non-palpable petechiae on the lower extremities with subsequent involvement of abdomen, lower back and buttocks. Abdominal exam showed diffuse tenderness without mass, guarding or rebound while reminder of physical exam was unremarkable. Following the failure of antimicrobial therapy, she was diagnosed with LCV by skin biopsy. Complete work up was negative for infectious, malignant and inflammatory etiologies of LCV. Patient improved with increased dose of budesonide and subsequently continued to tolerate ustekinumab without recurrence of LCV.

**Discussion and conclusion:**

LCV is a rare form of vasculitis and one of the rarest dermatologic manifestations of CD, appearing at any stage of the disease. LCV has been associated with autoimmune diseases, infections, specific drugs (levofloxacin, ustekinumab), and malignancy. Clinical presentation of LCV is variable and frequently mistaken for cellulitis. LCV should be considered in differential diagnosis of bilateral lower extremity rash in patients with CD after infectious, malignant and auto-immune/inflammatory etiologies are excluded. Unlike erythema nodosum (EN) and pyoderma gangrenosum (PG), LCV requires biopsy for diagnosis. Most patients respond well to steroids without scarring.

## Background

Inflammatory bowel disease (IBD) encompasses the two major entities of ulcerative colitis (UC) and Crohn’s disease (CD). Both diseases are chronic inflammatory disorders of poorly understood etiology, thought to occur in genetically predisposed individuals as result of an abnormal immunologic response to intestinal microbiome. UC lesions involve the mucosal and submucosal layers and are limited to the colon. CD lesions are transmural and typically involve the terminal ileum and colon, but may occur anywhere in the gastrointestinal tract from the mouth to perianal tissues with skipped areas of involvement [1]. Up to 40% of patients with IBD experience extra-intestinal manifestations (EIM), which occur more commonly in CD compared to UC. The most common EIM are musculoskeletal (peripheral arthritis), ocular (uveitis) and dermatologic [2]. The most common dermatologic manifestations of IBD are erythema nodosum (EN), pyoderma gangrenosum (PG) and aphthous stomatitis [3]. Less commonly seen dermatologic manifestations include neutrophilic dermatoses (Sweet’s syndrome), cutaneous vasculitis, epidermolysis bullosa acquisita, and metastatic CD [2, 3].

Leukocytoclastic vasculitis (LCV) is the rarest dermatologic manifestation of CD. Vasculitis is defined as a leukocyte-mediated inflammation of vessel walls, and may cause varying degrees of vessel destruction leading to hemorrhagic or ischemic events [4]. Vasculitides are classified by the size of blood vessels involved and by the dominant immune cells involved in the inflammation. LCV is an immune-mediated, small vessel vasculitis where inflammation is dominated by neutrophils, with fibrinoid necrosis and nuclear debris deposition (karyorrhexis) leading to post capillary venules damage [4]. The etiology of LCV is an abnormal immune response that may be precipitated by infections (e.g. viral hepatitis), medications (e.g. ustekinumab, levofloxacin), various malignancies, inflammatory/autoimmune diseases, or idiopathic stimuli [4, 5].

Here, we report a case of a patient with refractory CD whose clinical course was complicated by LCV, and discuss its challenging diagnosis in the context of previously reported cases.

## Case presentation

**Fig. 1:**
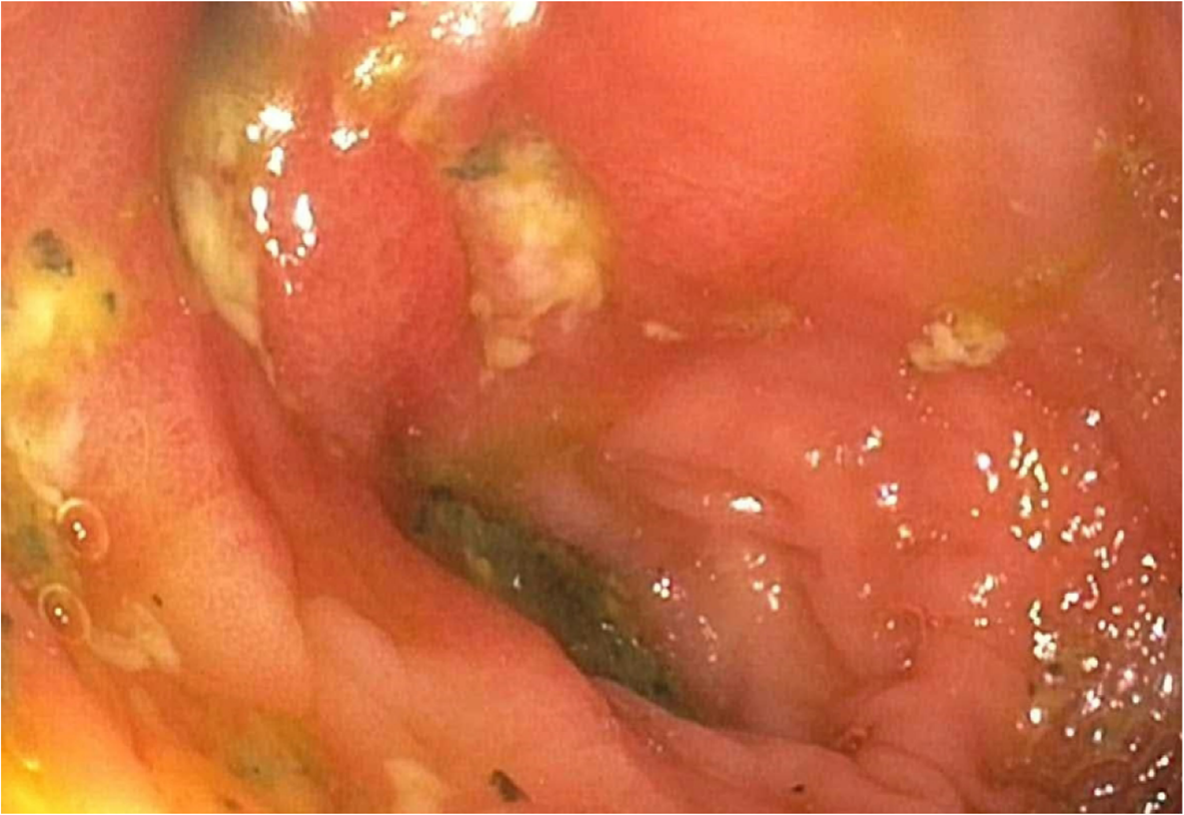
Findings from colonoscopy demonstrating hyperemic mucosa with superficial ulcerations

**Fig. 2:**
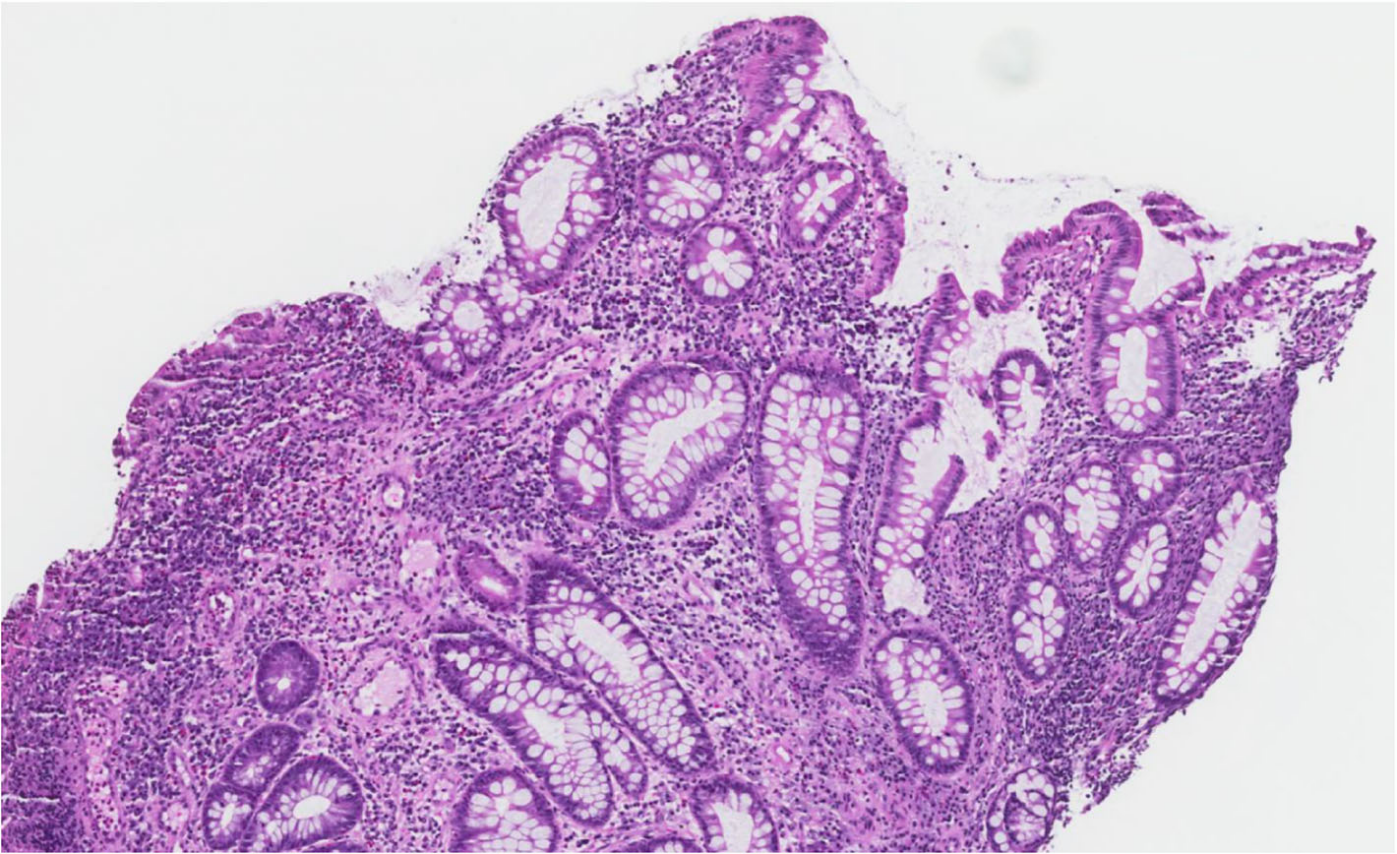
Biopsy of terminal ileum showing active chronic inflammation with ulceration consistent with Crohn’s disease (H&E stain, 40 x magnifications)

A 74-year-old woman, with a history of treatment-refractory Crohn’s disease on budesonide therapy (6 mg daily), psoriasis, and pernicious anemia developed abdominal pain, cramping, and non-bloody diarrhea 8 weeks prior to her current hospital admission. Colonoscopy was performed at onset of these flare of symptoms and demonstrated edema, erythema, and erosions at the ileocolic anastomosis (Fig. 1) with biopsies revealing severe active ileocolitis (Fig. 2). Standard dose of ustekinumab was added to her chronic budesonide regimen with moderate improvement in her symptoms. Approximately 6 weeks after ustekinumab was started and 2 weeks prior to her current admission, she was evaluated in urgent care with complaints of low-grade fever and a tender, non-pruritic bilateral lower extremity rash. At that time she began treatment with oral levofloxacin for presumed cellulitis. Her rash subsequently progressed, spreading to the abdomen and upper extremities. Her subjective fevers persisted, and she developed headache, fatigue, arthralgias, and generalized weakness along with worsening of abdominal pain and diarrhea. Given worsening of her symptoms despite antibiotic therapy, she presented to the emergency department for further evaluation and was admitted for presumed ineffective outpatient treatment of cellulitis and intravenous vancomycin therapy.

She was a retired factory worker from Wisconsin, USA who had been diagnosed with Crohn’s ileitis 30 years prior (A1, L3, B1) and maintained on budesonide. Prior therapies ineffective for her CD included mesalamine, prednisone, and adalimumab. Methotrexate trial was not tolerated due to allergic reaction (hives), and both infliximab and vedolizumab led to worsening of her psoriasis. Following the latest CD flare up standard dose of ustekinumab had been added to budesonide with the plan to continue the regimen with 8 week dosing intervals.

The patient was a former smoker who did not drink alcohol or use illicit drugs. Her medications included ustekinumab, budesonide, vitamin B12 injections and a multivitamin. Her psoriasis had been quiescent, and had required no recent treatment.

**Fig. 3:**
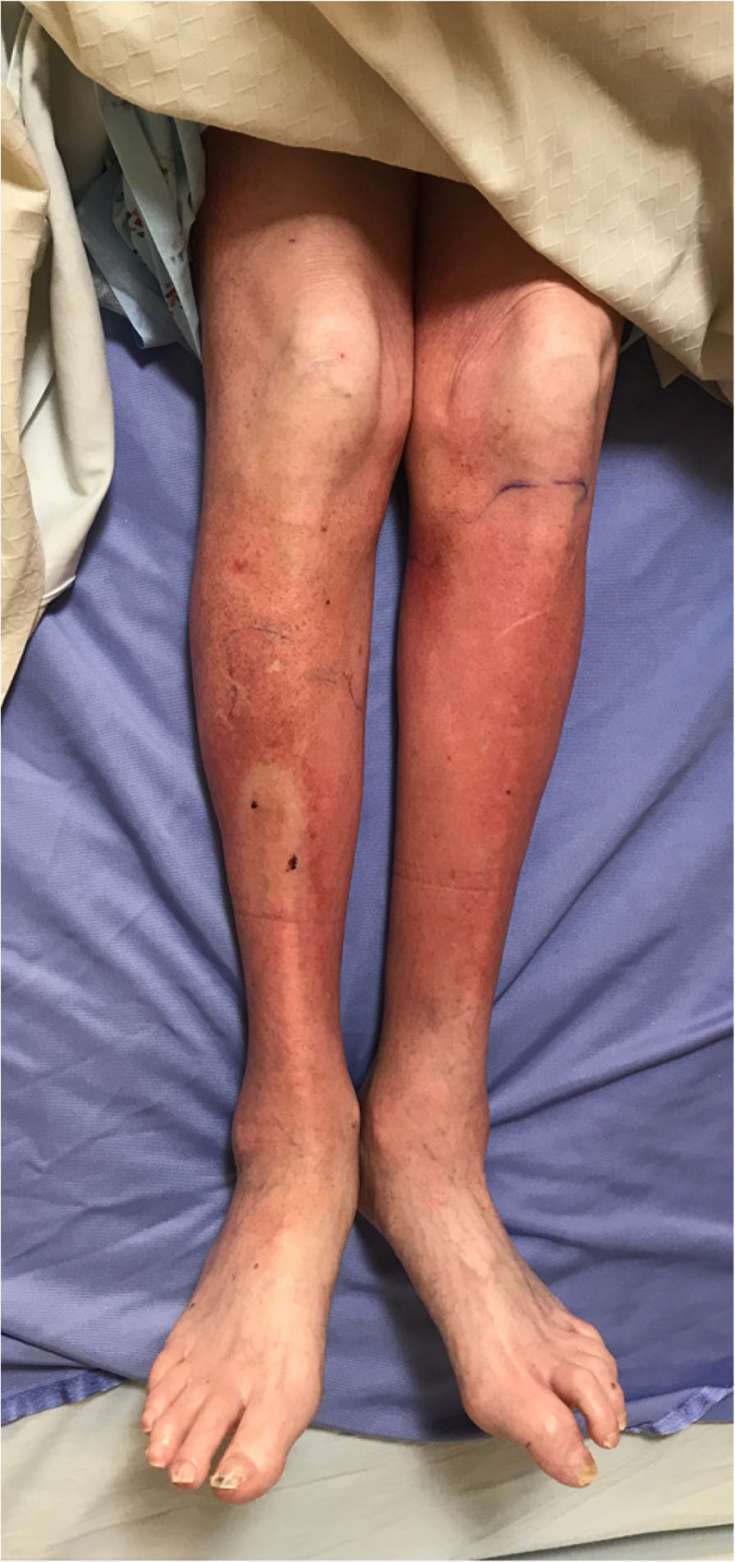
Tender macular erythematous rash involving the extensor surface of the lower limbs

On physical exam she appeared well-developed in mild distress due headache, abdominal pain, and painful rash. Patient was febrile to 38.2 °C; other vital signs were normal. Oral mucosa was moist and without ulcerations. Lungs were clear to auscultation bilaterally and heart sounds were regular, and without murmur. Abdominal exam revealed tenderness to deep palpation in the right lower quadrant without peritoneal signs. Rectal exam was negative for perianal fistulas. Neurologic exam was normal. Skin exam was remarkable for a tender, petechial rash involving her abdomen and extremities (Fig. 3).

A complete blood cell count demonstrated hemoglobin of 13.0 g/dL, white blood cell count of 5.7 x × 10^9^/L, and platelet count of 133 × 10^9^/L. A comprehensive metabolic panel demonstrated normal electrolytes, renal and liver function. Tick-borne panel including testing for Lyme disease, *Anaplasma spp*, *Ehrlichia spp* and *Babesia microti* was negative as well as blood cultures. Inflammatory markers were elevated with sedimentation rate (ESR) 42 mm/h, and C-reactive protein (CRP) 15.2 mg/L. Rheumatoid factor, cryoglobulins, and anti-nuclear antibody (ANA) were negative. Serum protein electrophoresis (SPEP) was negative for monoclonal protein spikes. Complement C3 and C4 levels were within normal range. Testing for viral hepatitis including Hepatitis A, B, and C were negative for both acute and chronic infection, and showed immunity against Hepatitis B virus. Cerebrospinal fluid (CSF) demonstrated a nucleated cell count of 0 with normal glucose and protein. A non-contrast head CT was negative for intracranial lesions. Recent mammogram and cervical cancer screening were normal.

**Fig. 4:**
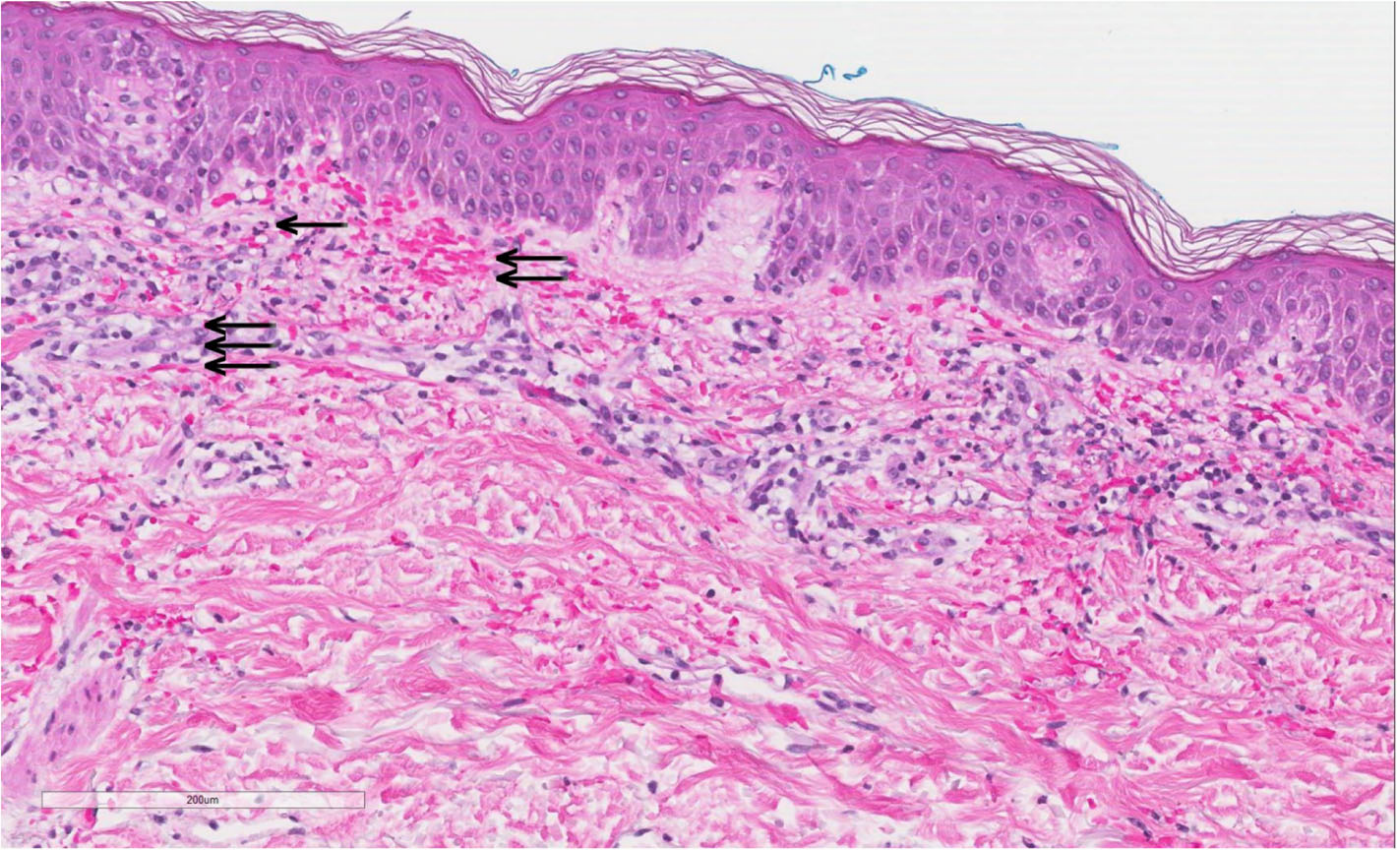
Skin biopsy (H&E stain,10x magnification) demonstrates inflammation with leukocytoclastic debris – karyorrhexis- (arrow), extravasated red blood cells (double arrow) and fibrinoid vascular degeneration (triple arrow) consistent with leukocytoclastic vasculitis

In the absence of leukocytosis and coupled with the symmetric distribution of the rash, cellulitis seemed unlikely, leading to discontinuation of antibiotics. Evaluation of rash with skin biopsy demonstrated findings consistent with leukocytoclastic vasculitis, including: 1) neutrophilic infiltration of the blood vessels with necrosis and perivascular fibrinoid deposition, 2) disruption of some small caliber blood vessels walls with hints of fibrin deposition, 3) bits of nuclear dust in and around blood vessel walls, and 4) extravasated erythrocytes (Fig. 4). A Periodic acid–Schiff stain (with appropriate positive control) did not demonstrate spore or hyphal form. Bacterial cultures of skin and blood remained negative.

High dose intravenous steroids were recommended as treatment of choice, but patient declined. Budesonide dose was subsequently increased from 6 mg to 9 mg daily, and within 48 h her rash began to subside, and patient noted improvement in abdominal pain and constitutional symptoms. Prior to discharge, she received another dose of ustekinumab without exacerbation of her rash, and with continued improvement in her gastrointestinal and constitutional symptoms. Eight months following her episode of CD flare associated with LCV she continued to do well on ustekinumab and budesonide has been gradually tapered off.

## Discussion and conclusion

Based on pathogenesis, IBD-associated skin disorders can be classified into the following groups: cutaneous granulomatous lesions with the same histological characteristics as IBD; reactive cutaneous manifestation due to shared antigens between gut microbes and the skin via antigen mimicry (such as with EN and PG); and dermatoses associated with IBD or its complications [3, 6]. Cutaneous manifestations of IBD are more common in females and particularly in those diagnosed at younger age and with family history of IBD [7–9].

Current postulates suggest that the rarest form of IBD-related skin disorders, LCV, is a reactive cutaneous manifestation [2, 7]. In spite of limited understanding of the pathogenesis of LCV in patients with IBD, several theories exist. One of the most plausible theories, the “theory of antigen mimicry,” postulates that certain epitopes of gut and skin flora are shared, very similar, or identical. Bacteria “leaks “through inflamed intestinal mucosa damaged by IBD and trigger an adaptive immune response. In some genetically predisposed individuals, the immune system is not able to discriminate between the epitopes of gut flora and those of the skin. Antigen-antibody immune complexes deposit in the skin, or in the case of LCV, within the small blood vessels of the dermis. This, in turn, causes inflammation and blood vessel destruction, erythrocyte extravasation, and manifestation of purpura. The proposed genetic predisposition may explain why some people develop EIM while others don’t. Data demonstrating presence of EIM in up to 84% of sibling pairs supports the genetic predisposition theory [9].

LCV most commonly presents as palpable purpura involving the lower extremities and may be pruritic or painful [7, 10]. It can, however, present in various forms such as erythema only, a petechial rash, a maculopapular rash, nodules, and various locations such as legs, abdomen or arms (Table 1). Similarly to other cutaneous IBD manifestations, LCV may occur independent of the disease activity of CD, yet in contrast to other skin manifestations of IBD manifestations, there are no reported cases of LCV preceding the CD diagnosis as it typically occurs either at the onset of CD or following its diagnosis. While PG can leave significant aesthetic sequela, LCV usually (but not always) resolves without scarring [10–21].

**Table 1:** Summarize previously published cases of LCV associated with CD

Case/Reference	Year of Publication	First Author	Patient age	Patient Gender	Etiology	Location of CD	Activity of CD	Location of rash	Therapy	Recurrence Y/N
1 [10]	1996	Zlatanic	39	M	CD	Transverse and sigmoid colon	Active	Legs	IV hydrocortisone	N
2 [11]	2002	McIlwain	24	F	Infliximab	Terminal ileum	Active	Legs and arms	Methylprednisolone	N
3 [12]	2008	Tsiamoulous	80	M	CD	Colon	Initial manifestation (active)	Legs	IV Prednisolone	N
4 [113]	2010	Limdi	52	M	CD	Ileum	Quiescent	Legs	IV steroids	N
5 [14]	2013	Karatoprak	28	M	CD	Jejunum	Initial manifestation (active)	Legs	Prednisone	N
6 [15]	2016	Namakura	35	M	Infliximab	NR	Quiescent	Legs	Spontaneous resolution	N
7 [16]	2017	Bernardes	29	F	Adalimumab	Colon	NR	Legs and abdomen	Discontinuation of Adalimumab	Y
8 [16]	2017	Bernardes	60	M	Adalimumab	Ileocolon	NR	Legs	Discontinuation of Adalimumab	N
9 [16]	2017	Bernardes	44	F	Adalimumab	Colon	NR	Legs	Discontinuation of Adalimumab	Y
10 [17]	2017	Goncalves	51	M	CD	Sigmoid and rectum	Initial manifestation (active)	Legs, thighs, arms	Prednisone	Y
11 [18]	2017	Woody	28	F	Certolizumab	NR	NR	Legs, feet, palms	Prednisone, Dapsone	N
12 [19]	2017	Cury	28	F	Adalimumab	NR	Quiescent	Legs	Colchicine	N
13 [20]	2018	Fonseca	38	F	Infliximab	Ileum	Quiescent	Legs	Prednisone	N
14 [21]	2019	Costa-Moreira	28	F	Ustekinumab	Perianal	Quiescent	Legs and abdomen	Prednisone	Y

*CD* Crohn’s Disease, *F* Female, *IV* Intravenous, *M* Male, *N* No, *Y* Yes, *NR* Not Reported

A PubMed database literature search for articles published in the English language including the key words “leukocytoclastic vasculitis” and “Crohn’s disease” yielded the 14 cases summarized in Table 1 [10–21].

In all identified cases represented in Table 1 and the case discussed herein, the rash affected the extensor surfaces of the bilateral lower extremities. In 35% of these 14 cases, the rash spread proximally to involve the abdomen and upper extremities. The patient in this case was initially misdiagnosed with cellulitis similar to the case reported by Limdi and Doran [13]. Both LCV and cellulitis display a similar constellation of constitutional signs and symptoms, fever, and rash. One important difference in these conditions to emphasize is that cellulitis rarely, if ever, presents in a bilateral distribution while vasculitis typically does [22]. Similar to this case, the majority of cases presented with a purpuric or petechial rash, which histologically corresponds to blood extravasation from small blood vessels due to vessel necrosis. While the clinical picture in the appropriate setting is sufficient for the diagnosis of PG or EN, skin biopsy remains the gold standard for LCV diagnosis and rules out infection or other causes of vasculitis.

Our patient developed LCV during an exacerbation of her CD. As shown in Table 1, a minority of cases of LCV were attributed directly to CD, while most cases (9 of 14, 62%) were attributed to the biological agents used to treat CD. Of 14 patients that we summarized in this review, 10 reported disease activity (e.g. quiescent vs. active). In five patients LCV developed during active CD (i.e. exacerbation) while the remaining 50% LCV occurred during clinically silent intestinal disease. While EN has been associated with disease activity, PG and LCV might or might not parallel intestinal disease activity [8, 9, 23]. Ileocolonic location of CD (as seen in our patient) has been associated with higher probability of development of EN or PG according to some authors [9, 24], however recent study of a Swiss IBD cohort did not demonstrate difference in disease location in patients who developed skin manifestation compared to those who did not [8]. In 40% of patients summarized in this review, approximately 40% (6 out of 14) had CD localized to large bowel.

Before attributing LCV as an EIM of IBD, it is important to exclude other etiologies that frequently accompany the disease, namely: medications, malignancy, connective tissue disorders, and infection. Given that steroids are the cornerstone of treatment for LCV, it is imperative that alternative causes of rash be thoroughly evaluated to avoid exacerbation of infection with steroid therapy.

Documented cases implicate both levofloxacin and ustekinumab in causing LCV [21, 25]. Levofloxacin as the cause of LCV in our case seemed unlikely given the presence of lesions prior to its administration. Ustekinumab was recently reported as a cause of vasculitis [21], adding it to the list of monoclonal antibodies implicated in LCV [11, 15, 16, 19, 20]. We considered ustekinumab as an etiology in this case, yet it seemed less likely when our patient tolerated re-initiation and long-term continuation of this medication without worsening of her LCV. While there is a possibility that the LCV was in fact due to ustekinumab and that higher doses of budesonide lead to clinical resolution, tapering off of budesonide with concurrent re-introduction of ustekinumab did not lead to reappearance of LCV. Thus, we can rule out medication induced LCV. As ustekinumab is a relatively new medication in armamentarium used to treat CD, it remains to be seen at what extent it might influence development of LCV. The only case report to date where LVC developed due to ustekinumab occurred after 3 years of therapy, while intestinal disease was in remission, and re-challenge with ustekinumab resulted in rapid re-appearance of LCV [21].

In our case, the patient’s recent and normal age-appropriate screening by mammogram, pap smear, and colonoscopy made malignancy an unlikely cause of LCV. Additionally, the extensive evaluation for other autoimmune disorders, connective tissue disease, and infection were unremarkable.

LCV responds well to steroid therapy and resolves quickly without scarring in most cases reported. Namakura [15] and colleagues reported a case of spontaneous resolution; Bernardes [16] reported 3 cases resolving without steroid therapy after withdrawal of adalimumab, and one case reported steroid-resistant LCV which resolved with colchicine [19]. While the rash subsides within days in the majority of cases, longer time to resolution has been reported [19]. The longest duration of rash reported was 9 months where infliximab was the inciting agent and no treatment or medication changes took place [19].

LCV is rare cutaneous manifestation of CD sometimes initially misdiagnosed as cellulitis. Clinicians should consider it in the differential diagnosis of patients with CD who present with constitutional symptoms and bilateral lower extremity rash. In approximately 30% of patients the rash can spread upwards to include abdomen, thorax and upper extremities and in more than 50% of reported cases it was associated with biologic therapy and not CD itself. In 50% of patients LCV occurred during CD exacerbation and 40% of patients had disease localized to the colon. Unlike other clinically diagnosable dermatologic manifestation of IBD, LCV requires a skin biopsy for diagnosis. In cases of suspected medication-induced LCV, appropriate management includes withdrawing the inciting medication. Systemic steroids show effectiveness for hastening recovery.

## Data Availability

All data are included within the manuscript

## Abbreviations

ANA: Anti-nuclear antibody
CD: Crohn’s disease
CRP: C-reactive protein
CSF: Cerebrospinal fluid
EN: Erythema nodosum
EIN: Extra-intestinal manifestations
ESR: Sedimentation rate
IBD: Inflammatory bowel disease
LCV: Leukocytoclastic vasculitis
NR: Not reported
PAS: Periodic acid-Schiff stain
PG: Pyoderma gangrenosum
SPEP: Serum protein electrophoresis
UC: Ulcerative colitis
